# Variation between glaucous and non-glaucous near-isogenic lines of rye (*Secale cereale* L.) under drought stress

**DOI:** 10.1038/s41598-022-26869-6

**Published:** 2022-12-28

**Authors:** Kamila Laskoś, Beata Myśków, Michał Dziurka, Marzena Warchoł, Kinga Dziurka, Katarzyna Juzoń, Ilona M. Czyczyło-Mysza

**Affiliations:** 1 The Franciszek Górski Institute of Plant Physiology Polish Academy of Sciences, Niezapominajek 21, 30-239 Kraków, Poland; 2 Department of Plant Genetics, Breeding and Biotechnology, West-Pomeranian University of Technology, Słowackiego 17, 71-434 Szczecin, Poland

**Keywords:** Plant physiology, Plant stress responses

## Abstract

Glaucous (811, L35, and RXL10) and non-glaucous (811bw, L35bw, and RXL10bw) near-isogenic lines (NILs) of rye (*Secale cereale* L.) forming three pairs of inbred lines were the subject of the research. The research aimed to study the relationship between wax cover attributes and the physio-biochemical drought reactions and yield of rye NILs and to uncover the differences in drought resistance levels of these lines. The greatest differences between glaucous and non-glaucous NILs were observed in the RXL10/RXL10bw pair. Of particular note were the stable grain number and the thousand grain weight of the non-glaucous line RXL10bw under drought and the accompanying reactions, such as an approximately 60% increase in MDA and a two-fold increase in wax amount, both of which were significantly higher than in the glaucous line RXL10 and in other NILs. The surprisingly high level of MDA in the RXL10bw line requires further analysis. Moreover, additional wax crystal aggregates were found under drought conditions on the abaxial leaf surface of the glaucous lines 811 and RXL10. The use of rye NILs indicated that line-specific drought resistance could be associated with wax biosynthetic pathways involved in physiological and biochemical responses important for increased drought resistance.

## Introduction

Winter rye (*Secale cereale* L.) is grown mainly in regions of central and eastern Europe. The global rye crop production in 2020 included 4.45 million ha and 15.02 million tons^1^. Rye can outyield wheat or barley in soil drought conditions on poor and medium soils^2,3^. Therefore, rye as plant material could be a valuable model to explain the functional and genetic basis of agronomic traits, which will also be significant in improving related Triticeae, bread wheat and barley^4^. The study of physiological and biochemical reactions in connection with the characteristics of the rye genome^5,6^ is necessary to obtain a complete picture of the performance of rye under abiotic stress conditions. Studying the effect of plant wax properties on yield and stress responses could open up new opportunities in cereals breeding^7–9^.

The primary determinant of plant growth and biomass production is photosynthesis, which provides the energy and carbon needed for the biosynthesis of organic compounds required during growth^10–12^. Photosynthesis is also one of the key processes affected by water shortages^13^. Drought in cereals leads to temporary limitation of the availability of water to the crop plant and influences the quantity and quality of crop yields. With a growing world population with increasing nutritional needs, drought periods that threaten the sustainability of cereal crops are becoming an increasing problem. Therefore, drought resistance became a particularly desirable trait among crops such as cereals^14^. It has been projected that future droughts may result in a further decrease in yields compared with current drought events^15^.

Wax bloom is one of the various adaptations that plants show to survive in adverse conditions^16^. Su et al.^17^ noted that wax attributes, as well as mesophyll and stomatal regulation, should be considered as an important factors in the breeding and selection of drought-resistant wheat cultivars. Similarly to wheat and barley, rye demonstrates a glaucous wax coating on above-ground plant organs^18^. In the context of abiotic stress, there is growing interest in the subject of epicuticular waxes, as evidenced by many reports describing the relationship between the cuticle and drought resistance^7,8,17,19–24^. The link between improved plant drought resistance and the role of genes and transcription factors in wax biosynthesis was shown in the literature^25–28^. The Arabidopsis study on MYB96 wax biosynthesis transcription factor^26^ suggested that during drought it may also be involved in other biosynthesis pathways, including those related to osmoprotectants and antioxidant biosynthesis. Interestingly, a study by Laskoś et al.^24^ on winter rye suggested a relationship between wax biosynthesis and the response of the plant to various environmental conditions and drought stress demonstrated by the correlation between leaf wax components and physiological and biochemical parameters of NILs, mostly chlorophyll *a* parameters, photosynthetic pigments and tocopherols. Cuticle thickness, composition, and morphology can change under soil drought stress^17^. In some plants, wax bloom appears as a trait easily distinguishable from the others, called glaucousness, manifested by a bluish coating on the above-ground organs. Glaucousness is a morphological characteristic of the epicuticular wax crystals that constitute the outermost layer of the plant cuticle^29^. The surface of the abaxial leaf blade, stem, and ear of glaucous cereal plants is characterized by the presence of tubular crystals, which are associated with increased synthesis of β-diketones and determine the glaucous phenotype^23,29–31^. This is a characteristic feature of Triticum species related to wax biosynthesis and conversion of very long-chain fatty acids; it requires activation of an additional β-diketone biosynthesis pathway^30,32^. The tubules can alter the optical properties of the plant surface, causing the structure to be perceived as a bluish glaucous appearance and leading to higher surface reflectance than crystals belonging to the platelet class^33,34^. The plant species, organ, and tissue determine the microstructure of epicuticular wax, which undergoes constant changes during ontogenetic development and may be modified by environmental factors^22,24,31,34,35^. Regarding essential cereals such as wheat (*Triticum aestivum* L.) or barley (*Hordeum vulgare* L.), the plant’s glaucousness is indicated as a positive attribute due to higher yield under water stress conditions^22,36,37^. Thus, it would be of great importance to explore whether glaucousness in the rye can be associated with water deficit resistance, which might be assessed through a comparison of the physiological and biochemical reactions of glaucous (GL) and non-glaucous (N-GL) plants under soil drought stress.

The presented research allowed for characterizing the reactions of rye plants to drought stress mainly in terms of their physiology [flag leaf of photosystem II (PSII), stomatal conductance and leaf greenness index], level of lipid peroxidation [malondialdehyde (MDA) content], wax crystal morphology and amount of flag leaf wax cover, and finally, relating these traits to plant yield and drought resistance indices. Based on the data mentioned above, the drought responses of each line were investigated and compared within each pair of NILs differing in wax cover type (GL versus N-GL). The use of unique plant material, such as near-isogenic GL and N-GL lines constituting three pairs, provided valuable information on the performance of photosynthetic apparatus and the yield of plants with different wax bloom. Also, changes in wax morphology during the water deficit were investigated. As an extension of our previous work (Laskoś et al. 2021), the additional pair of rye NILs consisting of GL RXL10 and N-GL RXL10bw lines were used, which exhibited more pronounced differences in reactions to drought than two other NIL pairs. The analysis was enhanced through the measurement of stomatal conductance, as well as the calculation of indicators of drought resistance. The study investigated differences in drought resistance of GL and N-GL rye lines and determined the relationship between wax cover attributes and the reactions to drought in rye NILs.

## Materials and methods

### Plant materials

Unique near-isogenic lines (NILs) of rye (*Secale cereale* L.) were obtained by the Department of Plant Genetics, Breeding, and Biotechnology of the Faculty of Environmental and Agricultural Sciences of the West Pomeranian University of Technology in Szczecin. The research material consisted of six inbred rye lines, three GL lines, and three N-GL lines (marked with the suffix "bw"), forming pairs of NILs: 811\811bw, L35\L35bw, and RXL10\RXL10bw. The N-GL lines (811bw; L35bw; RXL10bw) carry a recessive mutation that disrupts proper wax bloom formation; the plants appear waxless (the stems, leaves and ears are intensely green). In the pair of NILs, a typical GL line (811; L35; RXL10) was characterized by the presence of a waxy coating with a bluish tinge in the aboveground parts of the plant (glaucousness). The pedigree of NILs 811\811bw and L35\L35bw was described before^24^. The inbred line RXL10 originated from the Zeeland cultivar. The divergence into sublines (NILs) RXL10\RXL10bw occurred in a generation S_16_ and was caused by spontaneous mutation that disrupted the typical wax production, resulting in the appearance of N-GL plants. Sublines 811 and 811bw are lines with standard height and narrow leaves, while line pairs L35 and L35bw and RXL10 and RXL10bw are lines with a dwarf phenotype and broad leaves. The authors confirm that all methods used were performed in accordance with the relevant guidelines and legislation.

### Experimental design

A pot-based experiment was performed to evaluate the effect of drought stress on physiological and biochemical parameters and the yield of rye NILs. It was carried out in a semi-open vegetation tunnel protected from rain at *The F. Górski* Institute of Plant Physiology Polish Academy of Sciences, Kraków, Poland, and took place during the spring–summer period. Initially, the seeds of rye NILs were subjected to a 10-week vernalization in perlite at 4 ± 1 °C, then the seedlings were planted individually in pots. The containers (V = 3 l) were filled with a mixture of horticultural soil with a peat substrate and sand (1:1 v/v) of approximately 2.5 kg. The NILs (20 plants/line) were grown in a semi-open vegetation tunnel. Plant vegetation occurred under natural conditions of day and night length and temperature that coincided with the growth and development period of rye. At first, all plants grew under optimal soil moisture conditions, but once they reached the shooting stage, they were divided into groups of control and drought that differed in soil watering. Each group within a line consisted of 10 replications. The control group was watered daily with 50–200 ml of water per pot, while water withholding was applied in the drought group for 21 days. Volumetric measurements of soil water percentage were controlled during the experiment using a HydroSense® Soil Water Measurement System 620 (CAMPBELL SCIENTIFIC Inc., Shepshed, Leicestershire, UK) equipped with two 12-cm longitudinal probes attached to the sensor head. The following physiological measurements were made on the flag leaves of the main shoot on the last day of the drought period: chlorophyll *a* fluorescence kinetics, leaf greenness index, and stomatal conductance. The wax layer was then collected by washing the cut flag leaves with dichloromethane in test tubes. After wax collection, the flag leaves were frozen to calculate leaf hydration and further lipid peroxidation analysis. Furthermore, on the last day of drought, flag leaves from both treatments were sampled to examine the microstructure of the outer layer using a scanning electron microscope. After taking measurements and collecting samples, all plants were watered with the same amount of water (50–200 ml). At full maturity, the plants were harvested, then thousand grain weight (TGW) and drought resistance indices were determined.

### Scanning electron microscope (SEM) analysis of flag leaf surface

Scanning electron microscope (SEM) analysis of leaf surface of rye NILs was performed at using a HITACHI S-4700 microscope with a NORAN Vantage microanalysis system (Laboratory of Scanning Electron Microscopy and Microanalysis, the Institute of Geological Sciences, Jagiellonian University). Leaf samples of approximately 5 mm × 5 mm in size were collected on the last day of drought and then taped with carbon tape onto metal discs. As the SEM procedure requires water-free samples, the samples were air-dried and stored in a desiccator. The samples were sputtered with gold and analysed under a SEM, where micrographs of the upper and lower sides of the leaf blade were taken at a magnification of 10,000 × . In total, 24 samples were analysed [2 leaf samples (adaxial/abaxial leaf surface) × 2 treatments (drought and control) × 6 lines (811, 811bw, L35, L35bw, RXL10, RXL10bw)].

### Wax extraction and quantification

The flag leaves of 6–9 plants for each treatment (drought and control) within the line were cut after completion of physiological measurements. The wax layer was extracted by dipping the leaves 5 times in CH_2_Cl_2_ in a glass tube. A separate set of glass tubes was prepared, rinsed with CH_2_CH_2_ and then placed in a desiccator for 7 days to remove additional moisture from the tubes. The prepared tubes were weighed. The wax samples were filtered to a previously prepared set of glass tubes. After evaporation of CH_2_CH_2_ in an atmosphere of N_2_, the wax samples were placed back in the desiccator for 7 days to stabilize the weight. The wax fraction tubes were reweighed. The leaf wax load was calculated on the basis of differences between the weight of empty tubes and the weight of the wax tubes and converted to milligrams of leaf dry weight (DW). The samples were weighed with 0.001 mg precision on a semi-micro analytical balance (MYA 31.4Y; RADWAG, Radom, Poland).

### Flag leaf water content

After wax extraction, remaining leaf tissue was collected to a separate set of plastic tubes. The tissue was weighed to obtain fresh weight (FW), lyophilized for 72 h, and reweighed to obtain dry weight (DW). The following formula was used to calculate the leaf water content and was expressed as the percentage leaf hydration (LH): LH = [(FW-DW)/FW] × 100%.

### Physiological measurements

#### Measurement of the photochemical activity of PSII

Chlorophyll *a* fluorescence kinetics (CF) was measured using a Handy PEA fluorimeter (Hansatech, Kings Lynn, UK). Measurement was taken after shading the middle part of the leaf for 20 min using a clip. Selected CF parameters were calculated and analysed based on measurements of the OJIP test^38^. Measurements were made in five replicates for each treatment (drought and control) within a line. Based on the CF measurements, the following parameters per excited cross section (CS_m_) were measured and analysed as follows: F_v_/F_m_—the maximum photochemical efficiency of photosystem II (PSII); Area—the size of the electron acceptor field of PSII; PI—the overall performance index of PSII; ABS/CS_m_—the photon flux absorbed by antenna dye molecules; TR_o_/CS_m_—the excitation energy trapped in PSII reaction centers; ET_o_/CS_m_—the electron transport rate by PSII; DI_o_/CS_m_—the energy dissipated from PSII; RC/CS_m_—the density of active reaction centers.

#### Leaf greenness index

Noninvasive optical measurement of leaf greenness index (LGI) in the flag leaf was performed with a leaf chlorophyll meter SPAD 502 (Konica Minolta Sensing Europe B.V., Warrington, UK). The leaf was placed under the open measuring clip and slightly compressed. The device measured the difference between the absorption of light by leaf chlorophyll at a wavelength of 650 nm and the light absorbed by the other elements of the structure at 940 nm. The result were displayed in SPAD units, which were proportional to the chlorophyll content in the tested leaf and allowed for noninvasive comparing of plants. Measurements were made in five replicates for each treatment (drought and control) within a line.

#### Leaf stomatal conductance

The measurement of leaf stomatal conductance (*g*_s_) [(H_2_O) mmol·m-2·s-1] was performed using an AP4 porometer (Delta-T Devices Ltd, Cambridge, UK). *Stomatal conductance* was measured in the middle part of the flag leaf by placing it inside the measuring head of the device. The measurement was based on a comparison of the precisely measured humidity changes inside the measuring head with the readings obtained using the calibration plate. Measurements were made in five replicates for each treatment (drought and control) within a line.

#### Lipid peroxidation analysis (malondialdehyde quantification)

Malondialdehyde (MDA) content was measured spectrophotometrically according to Heath and Packer^39^. Lyophilized leaf tissue was ground in a mixing mill at the frequency of 30 Hz (MM 400; Retsch, Kroll, Germany). Then, the accurately weighed samples were extracted with 10% trichloroacetic acid (TCA), and then the homogenate was centrifuged. The supernatant was mixed with 0.5% thiobarbituric acid (TBA) in 10% TCA. After 30 min of incubation at 95 °C, the samples were transferred to 96-well microtiter plates. Absorbance was read at 532 nm using a microtiter plate reader (Synergy II, BioTek Instruments, Winooski, VT, USA). Pure malondialdehyde was used as a standard. Oxidative damage (lipid peroxidation level) was estimated based on the content of the MDA per milligram of leaf DW (µg/mg DW).

### Yield-related drought resistance indicators

Seeds were harvested at full maturity stage, and the weight of grains per plant (GW) as well as the thousand grain weight (TGW) expressed in grams were measured. Obtained values were used to calculate the drought to control percentage ratios (D/C) using following formula:

$$\frac{D}{C}=\left(\frac{D}{C}\right)\times 100\mathrm{\%},$$

where *C* was the average GN, GW or TGW for a given line in control conditions, *D* the average GW or TGW for a given line under drought conditions.

Based on the grain weight per plant following indexes were calculated (Table S2): drought susceptibility index (DSI)^40^, the tolerance index (TOL)^41,42^, mean productivity (MP)^41,42^, harmonic mean (HM)^43^, geometric mean productivity (GMP)^44^, yield index (YI)^45^, sensitivity drought index (SDI)^46^, yield stability index (YSI)^47^. Following formulas were used to calculate each of the respective indexes:

- $$DSI=\frac{1-\frac{{Y}_{D}}{{Y}_{C}}}{1-\frac{{\overline{Y} }_{D}}{{\overline{Y} }_{C}}}$$,
- $$TOL=$$ $${Y}_{C}-{Y}_{D}$$,
- $$MP=\frac{{Y}_{D}+{Y}_{C}}{2}$$,
- $$HM=\frac{2\times \left({Y}_{D}\times {Y}_{C}\right)}{{(Y}_{D}+{Y}_{C})}$$,
- $$GMP=\sqrt{{Y}_{D}\times {Y}_{C}}$$,
- $$YI=\frac{{Y}_{D}}{{\overline{Y} }_{D}}$$,
- $$SDI=\frac{{Y}_{C}-{Y}_{D}}{{Y}_{C}}$$,
- $$YSI=\frac{{Y}_{D}}{{Y}_{C}}$$,

where ${\mathrm{Y}}_{\mathrm{C}}$ is mean grain yield of each line in control, ${\mathrm{Y}}_{\mathrm{D}}$ is mean grain yield of each line under drought, ${\overline{\mathrm{Y}} }_{\mathrm{C}}$ is mean grain yield of all lines in control and ${\overline{\mathrm{Y}} }_{\mathrm{D}}$ is mean grain yield of all lines under drought.

### Data analysis

#### Statistical analysis

The calculation of means and their comparisons using Student's t-test, and principal component analysis (PCA) were performed using Statistica 13.1 software (StatSoft, Inc., Tulsa, OK, USA).

#### Analyses of biochemical and physiological responses of NILs to drought

To assess drought responses of the NILs, the drought to control percentage ratio (D/C) was calculated based on the obtained data for the CF parameters, stomatal conductance, leaf greenness index, malondialdehyde content, leaf hydration and wax amount. The following formula was used:

$$\frac{D}{C}=\left(\frac{D}{C}\right)\times 100\mathrm{\%},$$

where C was the average WA, LH, CF parameters, g_s_, LGI or MDA for a given line in control conditions, D the average WA, LH, CF parameters, g_s_, LGI or MDA for a given line under drought conditions.

#### PCA analysis of reactions to drought

To assess drought responses of the NILs in a joint analysis, the physiological, biochemical and yield responses to drought (D/C) and the yield-related drought resistance indices (DSI, TOL, MP, HM, GMP, YI, SDI, YSI) were analysed using the principal component analysis (PCA).

## Results

### Effect of wax cover type and drought on morphology of wax crystals on flag leaf surface

**Figure 1:**
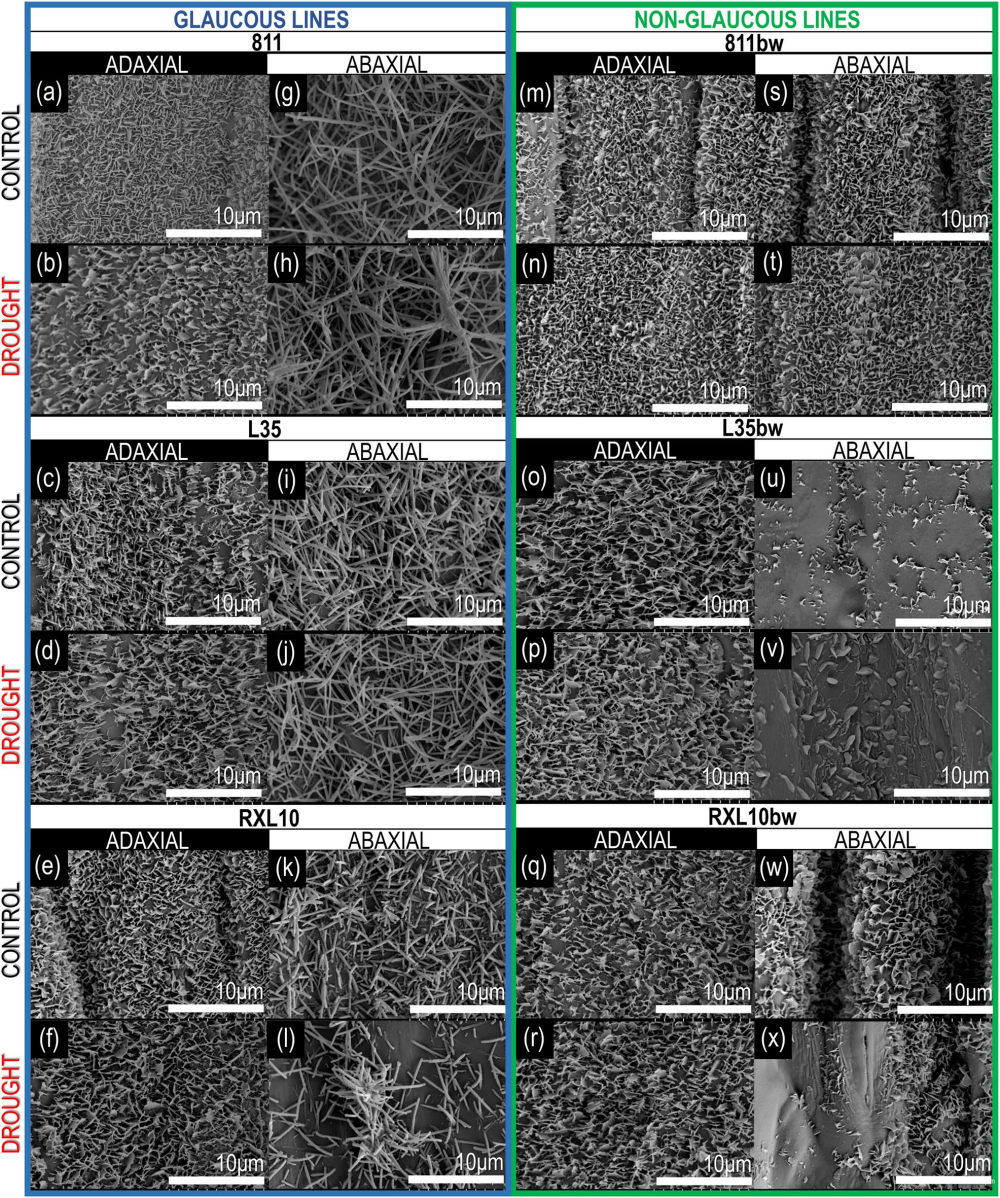
SEM images of wax crystals on the upper and lower leaf surfaces of near-isogenic rye lines: glaucous lines 811 (**a**,**b**,**g**,**h**), L35 (**c**,**d**,**i**,**j**) and RXL10 (**e**,**f**,**k**,**l**) and non-glaucous lines 811bw (**m**,**n**,**s**,**t**), L35bw (**o**,**p**,**u**,**v**) and RXL10bw (**q**,**r**,**w**,**x**) under control and soil drought stress conditions. 10 μm scale.

The SEM analysis of the adaxial and abaxial flag leaf surfaces of rye NILs demonstrated differences in the type of epicuticular wax crystals between the GL and N-GL lines (Fig. 1). According to the classification described by Barthlott et al.^29^, crystals of the tubular class were identified on the abaxial leaf surfaces of the GL lines(Fig. 1g–l) but were absent on the abaxial leaf surface of N-GL lines. Structures belonging to the irregular-edged platelet class were identified in N-GL lines (Fig. 1s–x). The adaxial surface of all examined leaves was covered with crystals from the asymmetrical plates group (Fig. 1a–f, m–r) and did not change under drought treatment; however, in the GL 811 line, it was less dense under drought conditions (Fig. 1b). The N-GL L35bw line was distinguished among all NILs, as the structures observed on the abaxial side under control conditions were small-scale, sparse crystals with irregular edges (Fig. 1u). Limited and thick structures with smooth edges were observed in L35bw treated with drought (Fig. 1v). A partial decrease in crystal alignment density was observed under drought conditions on the abaxial flag leaf surfaces of the GL RXL10 and N-GL RXL10bw lines (Fig. 1l,x). Also, in response to drought, the tubule clusters appeared on the abaxial leaf surface of the GL 811 and RXL10 lines (Fig. 1h,l). They took the form of extended clustering for the 811 line (Fig. 1h) and rosettes of tubules for the RXL10 line (Fig. 1l), respectively.

### Effect of wax cover type and drought on flag leaf hydration (LH) and wax amount (WA)

**Figure 2:**
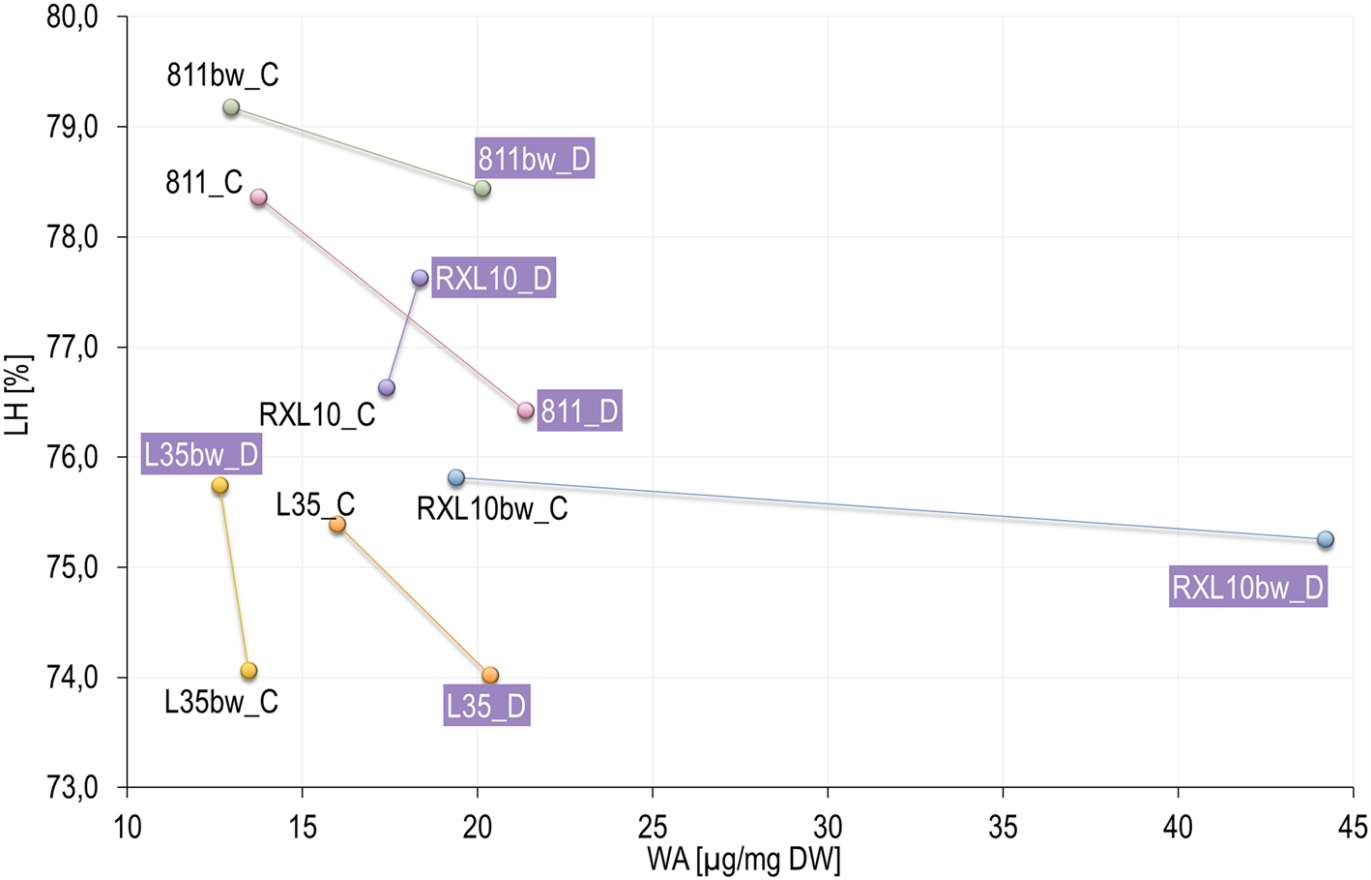
Changes in the mean percentage of flag leaf hydration (LH) and wax amount (WA) of near-isogenic glaucous (811, L35, RXL10) and non-glaucous (811bw, L35bw, RXL10bw) rye lines that constitute pairs 811/811bw, L35/L35bw, and RXL10/RXL10bw, under control (C) and drought conditions (D). Drought-treated objects are marked with purple rectangles.

The mean percentage of (LH) did not differ pronouncedly between treatments or between GL and N-GL lines in pairs (Fig. 2). LH ranged from 74 to 78% under both control and drought conditions, and the observed differences between these groups reached a maximum of 2.5% (Fig. 2). On the other hand, most lines, showed an increase in WA (up to 128%) under drought conditions, except for a slight decrease noted in the N-GL L35bw line (Fig. 2). In the pair of NILs 811 and N-GL 811bw, both lines demonstrated a similar wax cover gain of 55.5% (Fig. 2). However, the GL 811 line showed a higher wax load both in control and drought conditions by 0.8 and 1.2 µg/mg DW, respectively (Fig. 2). The N-GL L35bw line showed a loss of wax amount by 1 µg/mg DW (a 6.1% decrease) in response to drought, while the GL L35 line expressed an increase in wax load of approximately 4.0 µg/mg DW (a 27.3% increase) (Fig. 2). For this reason, the GL L35 demonstrated a higher wax load in both treatments, which was more evident under drought (Fig. 2). In response to drought, both GL RXL10 and N-GL RXL10bw lines showed increase in wax load, but the N-GL RXL10bw line revealed more than twice as much wax as the GL RXL10 line, and was distinguished among all NILs for having the highest WA increase (128%) and highest wax weight on the last day of drought (Fig. 2).

### Effect of wax cover type and drought on stomatal conductance (*g*_s_) of flag leaf

**Figure 3:**
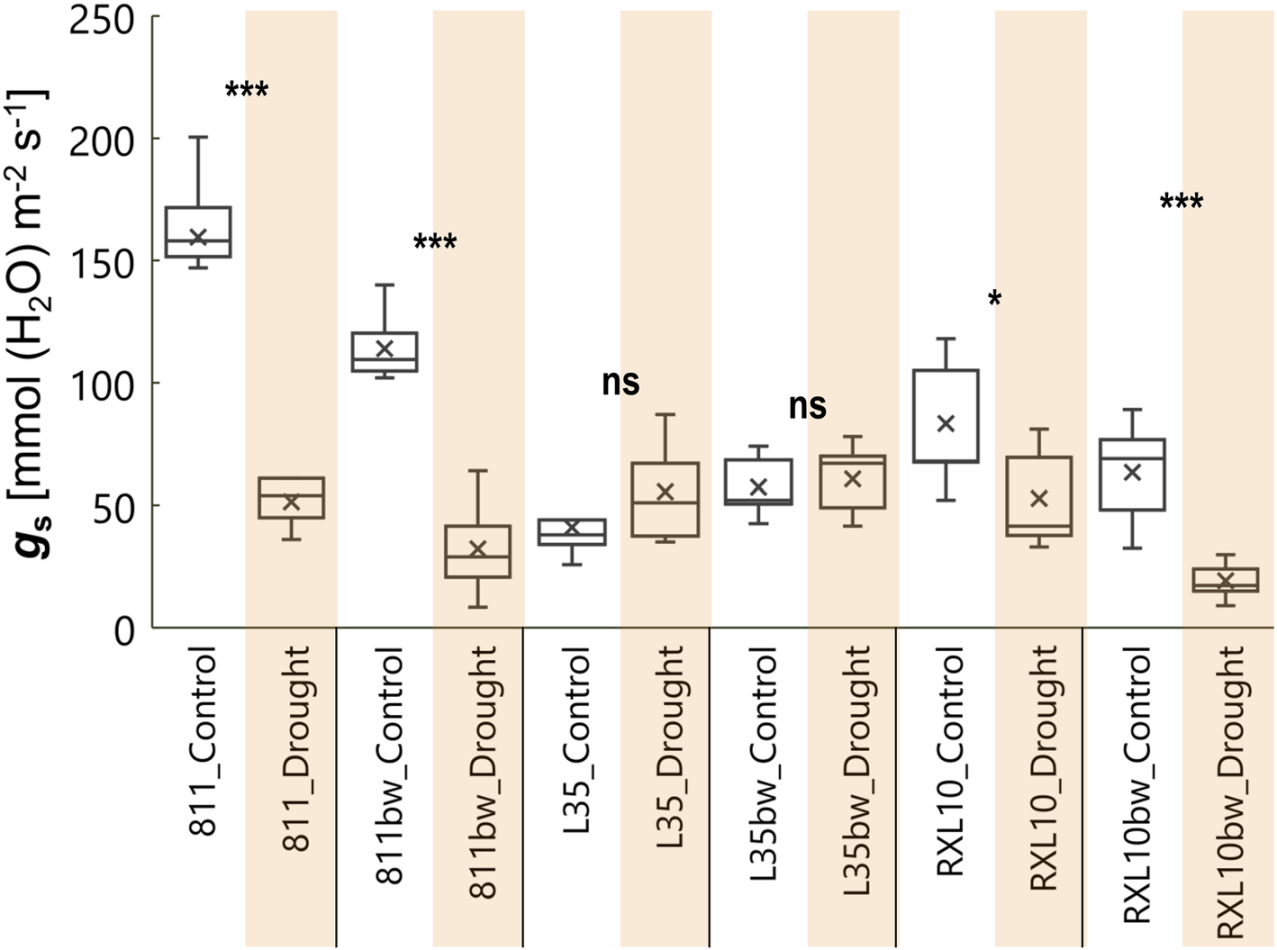
Box plot of leaf stomatal conductance (*g*_s_) by glaucous (811, L35, RXL10) and non-glaucous (811bw, L35bw, RXL10bw) near-isogenic lines of rye and by soil water status (control or drought). The statistically significant differences between the means in control and drought were verified for P ≤ 0.001(***), P ≤ 0.01(**), P ≤ 0.05(*) according to Student’s test. *ns* not significant.

Under control conditions, the 811/811bw NILs pair showed significantly higher *g*_s_ (112–162 mmol (H_2_O) m^−2^ s^−1^) than the other two pairs of NILs (41–80 mmol (H_2_O) m^−2^ s^−1^) (Fig. 3). As a result of drought, the *g*_s_ of the rye flag leaf decreased significantly in two pairs of NILs: 811/811bw and RXL10/RXL10bw (67.8–71.7%) (Fig. 3). The NILs of the L35/L35bw pair demonstrated an increasing trend in *g*_s_, but it was not statistically significant (Fig. 3). On the last day of drought, the lowest *g*_s_ (19.14 mmol (H_2_O) m^−2^ s^−1^) was shown by the N-GL RXL10bw line (Fig. 3).

### Effect of wax cover type and drought on chlorophyll *a* fluorescence (CF) parameters of flag leaf

**Figure 4:**
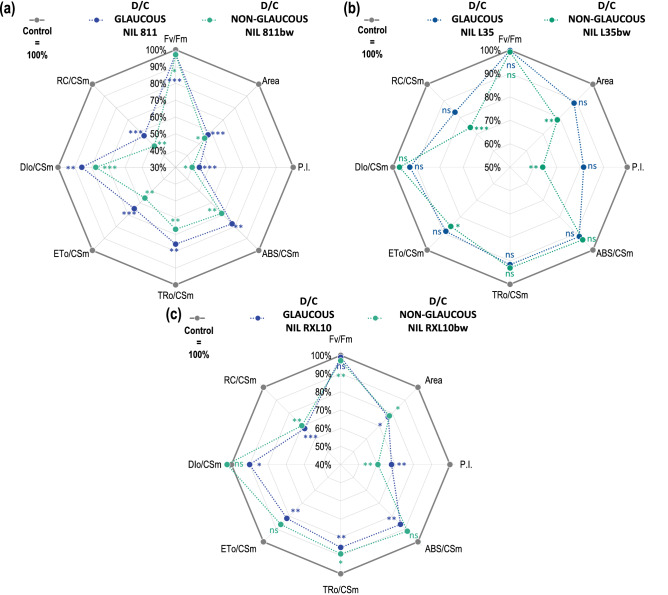
The drought to control percentage ratio (D/C) of chlorophyll *a* fluorescence kinetic parameters (F_v_/F_m_, Area, PI, ABS/CS_m_, TR_o_/CS_m_, ET_o_/CS_m_, DI_o_/CS_m_, RC/CS_m_) in glaucous (811, L35, RXL10) and non-glaucous (811bw, L35bw, RXL10bw) near-isogenic rye lines. The grey line states for 100% of each line (control), D/C was calculated from the following formula: %CD/C = (D/C) × 100%, where C is the average value of the CF parameter for a given line in control conditions, D is the average value of the CF parameter for a given line under drought condition. The statistically significant differences between the means in control and drought were verified for P ≤ 0.001(***), P ≤ 0.01(**), P ≤ 0.05(*) according to Student’s test. *ns* not significant, *F*_*v*_*/F*_*m*_ the maximum photochemical efficiency of photosystem II (PSII), *Area* the size of the electron acceptor field of PSII, *PI* the overall performance index of PSII, *ABS/CS*_*m*_ the photon flux absorbed by antenna dye molecules, *TR*_*o*_*/CS*_*m*_ the excitation energy trapped in PSII reaction centers, *ET*_*o*_*/CS*_*m*_ the electron transport rate by PSII, *DI*_*o*_*/CS*_*m*_ the energy dissipated from PSII, *RC/CS*_*m*_ the density of active reaction centers.

When chlorophyll *a* fluorescence (CF) was measured, the following parameters were determined: F_v_/F_m_, Area, PI, ABS/CS_m_, TR_o_/CS_m_, ET_o_/CS_m_, DI_o_/CS_m_, and RC/CS_m_ (Fig. 4, Table S1). Drought stress affected the condition of the photosynthetic apparatus by decreasing the values of the measured CF parameters in most GL and N-GL rye lines (Fig. 4). However, a significant decrease (up to 60%) in all CF parameters (except F_v_/F_m_) in response to drought was recorded only in the GL 811 and N-GL 811bw lines (Fig. 4a). The maximum photochemical efficiency of PSII (F_v_/F_m_) was not sensitive to drought compared with the other CF parameters, and the drought to control percentage ratio (D/C) is close to 100% (Fig. 4). Only slight decreases in F_v_/F_m_ were observed in the GL 811, N-GL 811bw and N-GL RXL10bw lines, and in the other NILs the value of the D/C for this parameter was close to 100% (Fig. 4). The pair of GL 811 and N-GL 811bw lines showed a lower D/C in case of the CF parameters (Fig. 4). Both NILs GL 811 and the N-GL 811 experienced a significant reduction in the area over the chlorophyll *a* fluorescence induction curve (Area) by 43% and 46%, respectively, and the overall performance index of PSII photochemistry (PI) by 56 and 60%, respectively (Fig. 4a). Although the differences between D/C in GL and N-GL NILs were slight (3–4%), the N-GL 811bw line showed a lower D/C of CF parameters related to light energy absorption (ABS/CS_m_) and its intake for electron transport (ET_o_/CS_m_), as well as excitation energy trapped in reaction centers (TR_o_/CS_m_), the number of active reaction centers (RC/CS_m_) and the DI_o_/CS_m_ parameter, explaining the amount of energy dissipated from PSII (equal to [ABS/CS_m_—TR_o_/CS_m_]) (Fig. 4a). The GL L35 and N-GL L35bw were characterized by a pronounced reduction in Area (11–22%), PI (19–36%), and RC/CS_m_ (17–26%) under drought conditions, which was particularly significant regarding the N-GL L35bw line (Fig. 4b). The drought caused a 7.3% decrease in the DI_o_/CS_m_ parameter in the GL L35 line, while a 2.8%reduction was reported in the N-GL L35bw line (Fig. 4b). In terms of ABS/CS_m_, ET_o_/CS_m_, and TR_o_/CS_m_, both the GL and N-GL lines showed a decline of approximately 6–14%, but the differences between the GL L35 and N-GL L35bw lines did not exceed 3% (Fig. 4b). Similarly, in the RXL10/RXL10bw pair, drought caused a significant reduction in Area, PI, and RC/CS_m_, which amounted to 22–23%, 32–40%, and 30–32%, respectively (Fig. 4c). Particularly evident differences between the reaction to the drought of GL and N-GL lines concerned the PI, which decreased in the GL RXL10 and N-GL RXL10bw line by 32% and 40%, respectively (Fig. 4c). Minor differences between the GL and N-GL lines were reported regarding the D/C of Area and RC/CS_m_ (Fig. 4c). The GL RXL10 and N-GL RXL10bw were characterized by a reduction in ABS/CS_m_, (13.6–8.2%), ET_o_/CS_m_ (14.5–10.9%), and TR_o_/CS_m_ (18.2–13.5%) under drought conditions, which was more pronounced regarding the GL RXL10 line (Fig. 4c). On the other hand, the GL RXL10 line showed a decrease of 10% in the amount of energy dissipated from PSII (DI_o_/CS_m_), while no significant change was observed in the N-GL RXL10bw line (Fig. 4c).

### Effect of wax cover type and drought on flag leaf greenness index (LGI)

**Figure 5:**
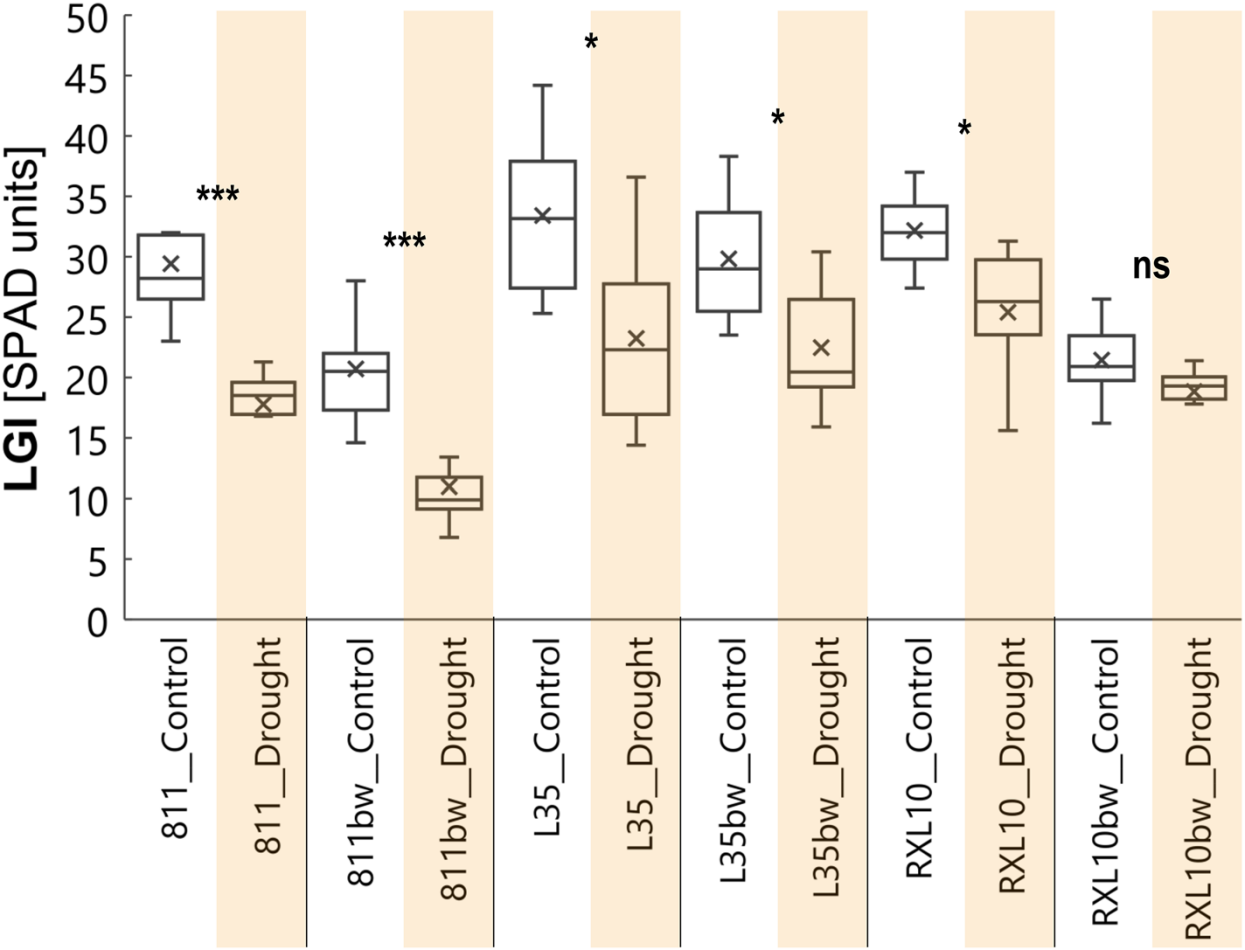
Box plot of leaf greenness index (LGI) by glaucous (811, L35, RXL10) and non-glaucous (811bw, L35bw, RXL10bw) near-isogenic lines of rye and by soil water status (control or drought). The statistically significant differences between the means in control and drought were verified for P ≤ 0.001(***), P ≤ 0.01(**), P ≤ 0.05(*) according to Student’s test. *ns* not significant.

Drought stress significantly reduced leaf greenness index (LGI) in all lines except the N-GL RXL10bw line (Fig. 5), which was consistent with the decrease in CF parameters (Fig. 4). The most substantial decline in LGI was observed in the GL 811 and N-GL 811bw lines, which were 39.5% and 47.0%, respectively (Fig. 5). Regarding the pair of NILs L35/L35bw under drought conditions, there were no pronounced differences in LGI between the GL (23.2 SPAD units) and N-GL (22.5 SPAD units) lines (Fig. 5). However, LGI reduction in response to drought differed between the GL L35 and N-GL L35bw lines and amounted to 30.4% and 24.6%, respectively (Fig. 5). In the case of the GL RXL10 and N-GL RXL10bw lines, there are visible differences between them in both treatments; however, LGI decreased significantly by 21% in response to drought in the GL RXL10 line, while a 12% decrease was reported in the RXL10bw line (Fig. 5).

### Effect of wax cover type and drought on malondialdehyde (MDA) content in flag leaf

**Figure 6:**
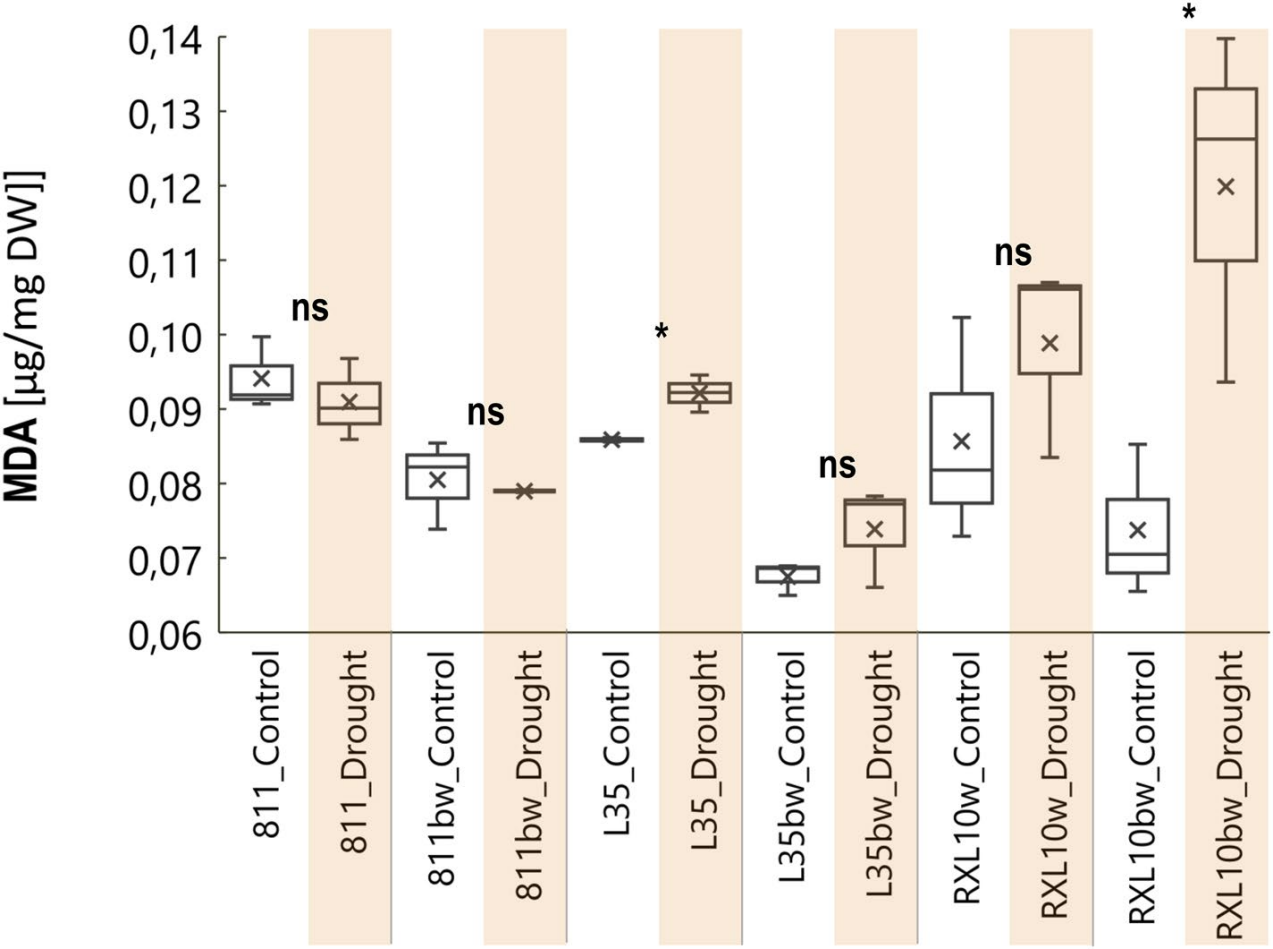
Box plot of malondialdehyde content (MDA) by glaucous (811, L35, RXL10) and non-glaucous (811bw, L35bw, RXL10bw) near-isogenic lines of rye and by soil water status (control or drought). The statistically significant differences between the means in control and drought were verified for P ≤ 0.001(***), P ≤ 0.01(**), P ≤ 0.05(*) according to Student’s test. *Ns* not significant.

Malondialdehyde (MDA) content under optimal conditions in GL lines was about 0.9 µg/mg DW, and in the N-GL lines—0.7–0.8 µg/mg DW (Fig. 6). The GL 811 and N-GL 811bw lines demonstrated a minor and insignificant decrease in leaf MDA level (1–3%) (Fig. 6). During drought conditions, a significant increase in flag leaf MDA was observed in the GL L35 and N-GL RXL10bw lines; however, regarding the L35 line, the increase was slight (0.006 µg/mg DW (Fig. 6). The N-GL RXL10bw line demonstrated the most substantial effect of drought on the MDA content, which increased from 0.07 to 0.12 µg/mg DW (Fig. 6). The MDA content did not change significantly in the 811, 811bw, L35bw, and RXL10 lines (Fig. 6).

### Effect of wax cover type and drought on thousand grain weight and grain number

**Figure 7:**
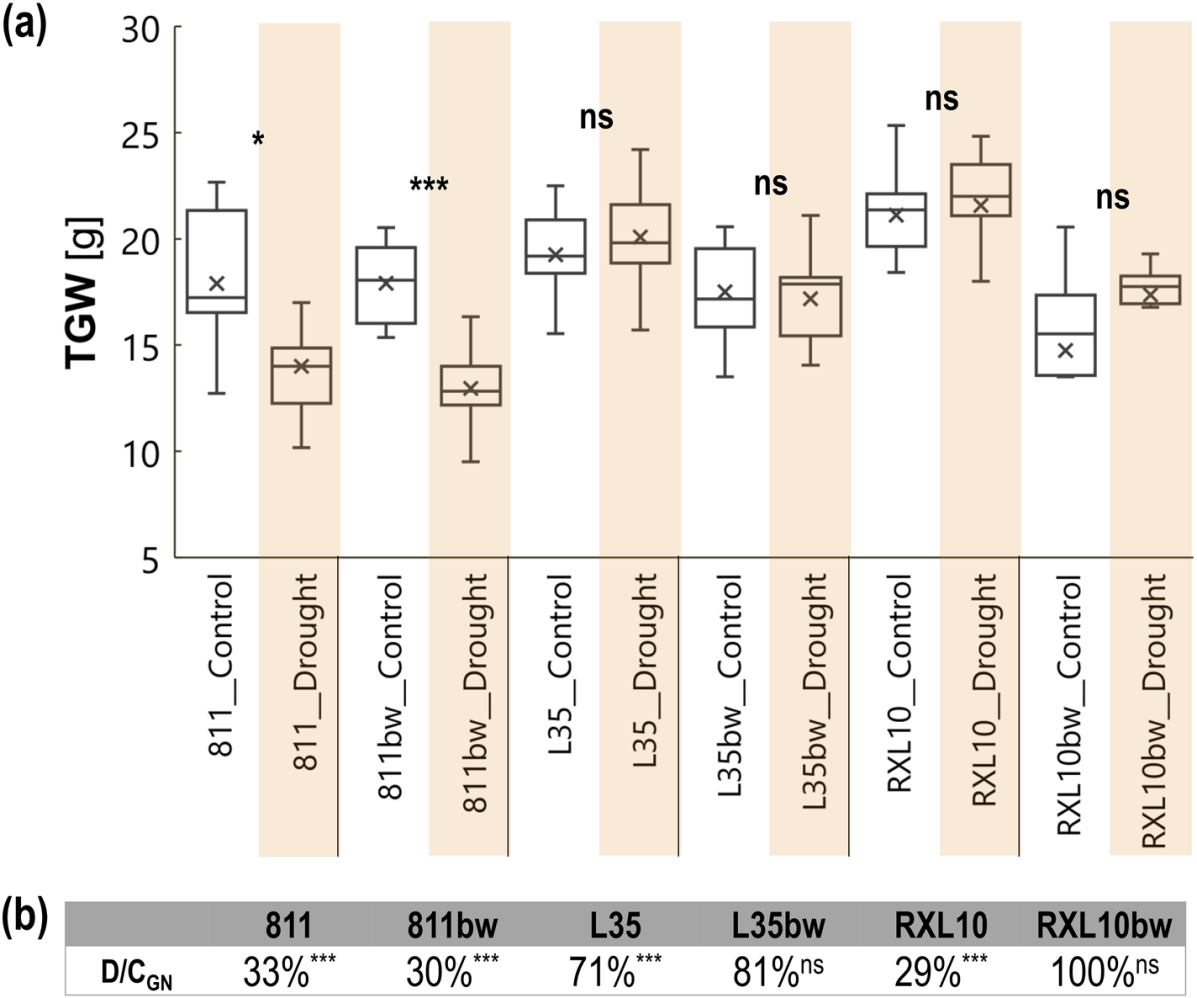
Box plot (**a**) of leaf thousand grain weight (TGW) by glaucous (811, L35, RXL10) and non-glaucous (811bw, L35bw, RXL10bw) near-isogenic lines of rye and by soil water status (control or drought) and the drought to control percentage ratio (**b**) of grain number (D/C_GN_) in glaucous (811, L35, RXL10) and non-glaucous (811bw, L35bw, RXL10bw) near-isogenic rye lines. The statistically significant differences between the means in control and drought were verified for P ≤ 0.001(***), P ≤ 0.01(**), P ≤ 0.05(*) according to Student’s test. *Ns* not significant.

The GL lines 811, L35 and RXL10 revealed a higher thousand grain weight (TGW) under drought conditions than their N-GL counterparts by 1.1, 2.9 and 4.2 g, respectively (Fig. 7a). Long-term water deficit caused a significant decrease in TGW of the GL 811 and N-GL 811bw lines (Fig. 7a), which was also accompanied by a substantial drop in grain number expressed as low values (33% and 30%, respectively) of D/C_GN_ (Fig. 7b). In the case of D/C, the GL 811 and N-GL 811bw lines showed a similar decrease in seed number in response to drought (Fig. 7b), while in the case of TGW, drought more significantly reduced TGW in the N-GL 811bw line (a 27% decrease in the N-GL line versus a 21% decrease in the GL line) (Fig. 7a). TGW The other NILs appeared to be more stable in terms of yield under drought conditions in terms of TGW (Fig. 7). Interestingly, for TGW the N-GL RXL10bw line, both the TGW and D/C were stable in response to drought (Fig. 7). On the contrary, stable TGW in the GL RXL10 line under drought (Fig. 7a) was accompanied by a significant decrease in seed number (D/C_GN_ = 29%) (Fig. 7b). The GL L35 and N-GL L35bw lines experienced a grain number decrease of 29% and 19%, however it was significant only for the GL L35 line (Fig. 7b).

### Correlation between yield-based drought resistance indices and physiological-biochemical changes under drought in near-isogenic lines differing in wax cover type

**Figure 8:**
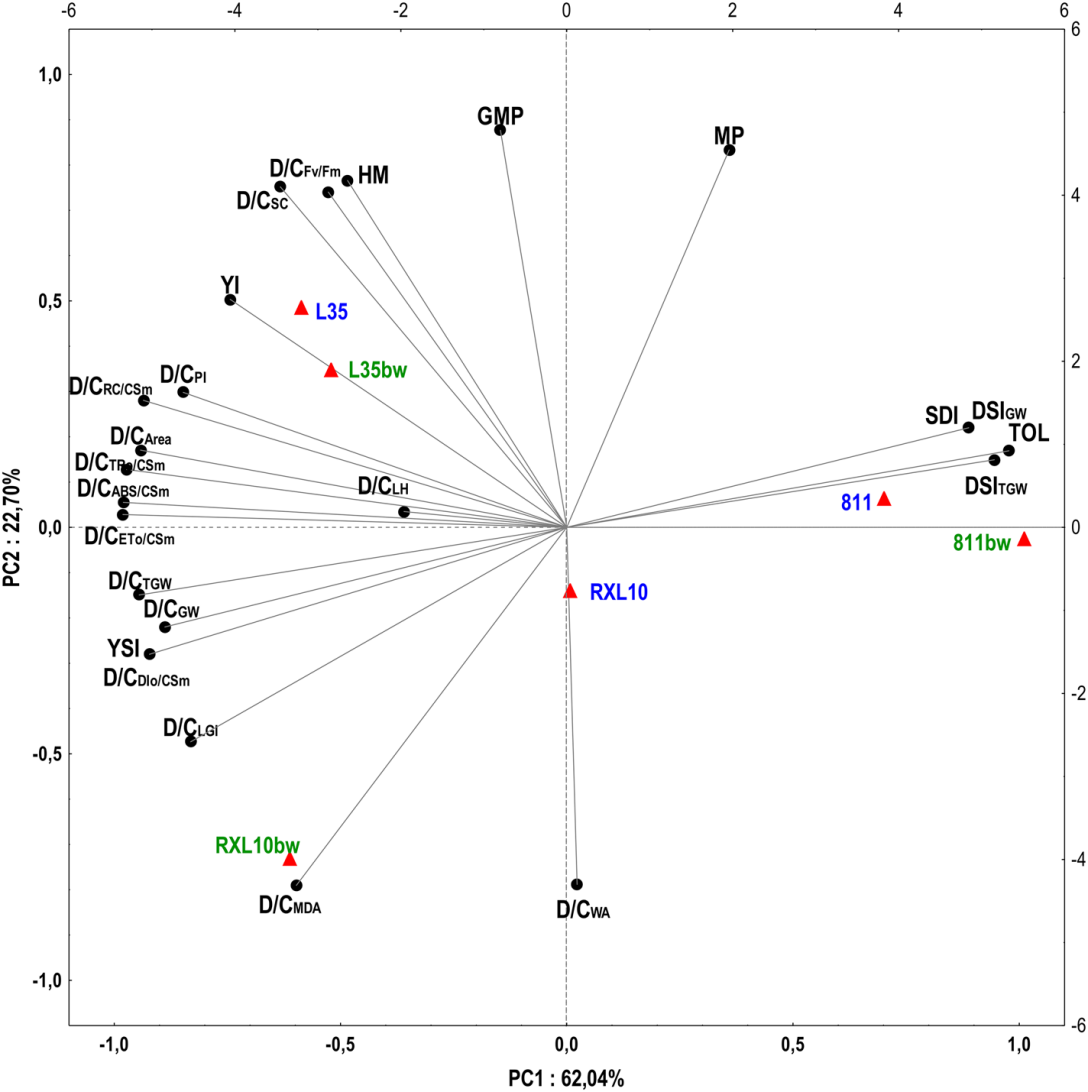
Principal component analysis (PCA) presented as a biplot (projection of cases and projection of variables) on the plane of the first two factor axes (PC1, PC2) performed for variables differentiating the studied near-isogenic glaucous (811, L35, RXL10; marked in blue) and non-glaucous (811bw, L35bw, RXL10bw; marked in green) rye lines in terms of physiological and biochemical responses to soil drought and yield. The following variables were analysed: the drought to control percentage ratio (D/C) of chlorophyll fluorescence kinetic parameters (F_v_/F_m_, Area, PI, ABS/CS_m_, TR_o_/CS_m_, ET_o_/CS_m_, DI_o_/CS_m_, RC/CS_m_), stomatal conductance (*g*_s_), leaf greenness index (LGI), malondialdehyde (MDA) content, leaf hydration (LH), wax amount (WA) and yield-related drought resistance indices, i.e., the drought to control percentage ratio (D/C_GW_ and D/C_TGW_) and Drought Susceptibility Index (DSI_GW_ and DSI_TGW_) calculated based on grain weight (GW) and thousand grain weight (TGW), and indices calculated based on grain weight, i.e. tolerance index (TOL), mean productivity (MP), harmonic mean (HM), geometric mean productivity (GMP), Yield Index (YI), Sensitivity Drought Index (SDI), Yield Stability Index (YSI).

According to the principal component analysis (PCA), principal factors 1 (PC1) and 2 (PC2) explained a total of 84.74% of the variation between the studied NILs in terms of their response to soil drought stress (Fig. 8). PC1 explained 62.04% of the variability, which was most significantly shaped by the drought to control percentage ratio (D/C) of chlorophyll *a* fluorescence parameters associated with phenomenological flows (ABS/CS_m_, TR_o_/CS_m_, ET_o_/CS_m_), and drought stress indices (DSI_GW_, DSI_TGW_, D/C_GW_, D/C_TGW_), tolerance (TOL), yield stability indices (YSI), and drought sensitivity indices (SDI) (Fig. 8). Within PC2, the parameters D/C_Fv/Fm_, D/C_MDA_, D/C_*g*s_, D/C_WA_, MP, and GMP explained 22.70% of the variation between the tested lines (Fig. 8). The PCA analysis highlighted correlations among the indicators that explain the variance among tested NILs (Fig. 8). The most prominent relations revealed by these biplot are: (i) strong negative correlations (large obtuse angle) between the D/C of the CF parameters and the yield-related indices (TOL, DSI and SDI), (ii) strong positive correlations (acute angles) between the D/C of the CF parameters and the YSI, D/C_TGW_ and D/C_GW_ (Fig. 8). The different arrangement of the NILs on the biplot relative to the vectors indicates the differentiation of the tested rye NILs in terms of physiological and biochemical parameters and yield in response to drought conditions (Fig. 8). On the other hand, the NILs arrangement showed a slight separation of the GL and N-GL lines in the 811/811bw and L35/L35bw pairs, while the GL RXL10 and N-GL RXL10bw constituting a NILs pair were located in distant parts of the coordinate system (Fig. 8). Therefore, a clear difference between the GL and N-GL lines regarding the analysed drought resistance indexes was observed only in NILs pair RXL10/RXL10bw (Fig. 8). The position of the L35/L35bw NIL pair and the N-GL RXL10bw line in the biplot coordinate system indicate higher D/C values of chlorophyll *a* fluorescence parameters, higher yield stability (YSI) and lower values of the drought susceptibility parameter (SDI), tolerance index (TOL), DSI_GW_, and DSITGW compared with the GL RXL10 line and the 811/811bw NIL pair (Fig. 8). The N-GL RXL10bw line was clearly distinguished from the other NILs, including its counterpart, the GL RXL10 line, in terms of wax accumulation (D/C_WA_) and also MDA level (D/C_MDA_) (Fig. 8).

## Discussion

Plants show a variety of morphological, physiological, and biochemical responses to drought stress and use different adaptive mechanisms, including wax accumulation^17,22,48^ According to Xue et al.^27^, a comprehensive study is of high importance, especially the physiological and agronomical interrelationships between the attributes of cuticular wax (structure and chemical composition) and drought stress. The subject of our research was studying GL and N-GL plants constituting pairs of NILs, characterised by different wax ultrastructure on above-ground organs, i.e. leaf, stem, and abaxial leaf surface. We investigated the connection between the wax structure (GL versus N-GL) that covers the green parts of rye and physiological and biochemical responses to soil drought stress.

### Effect of wax cover type and drought on morphology of wax crystals on flag leaf surface

SEM examination of flag leaf surfaces allows for rapid comparing in terms of the effects of soil drought on leaf surface ultrastructure, but depending on species, cultivar and type of wax cover, these responses may be different. In the current study, the tubular and platelet classes of ultrastructure were identified on the abaxial and adaxial leaf surface of the rye flag leaf in the GL and N-GL lines. It was consistent with the crystal structures previously identified in rye NILs^24^, as well as in GL and N-GL wheat NILs^17,22^. The present investigation highlighted the spatial modifications in the microstructure of the epicuticular waxes of the GL 811 and RXL10 lines treated with drought (Fig. 1h,l), not detected during the study of line 811 by Laskoś et al.^24^. The tubular-shaped crystalline rosette clusters on the abaxial surface of the GL RXL10 line were similar to those imaged on the abaxial surface of GL wheat lines in the study by Su et al.^17^. The abaxial leaf surface of the N-GL L35bw line under drought developed much smaller structures of indefinite shape as in Laskoś et al.^24^, which is also consistent with Guo et al.^22^ research on the leaf surface of N-GL wheat lines. Various changes in the density of epicuticular wax crystals were reported in cereals under stress factors^7,17,22,49,50^. The leaf surface exhibits substantial heterogeneity in optical properties determined by species and environmental conditions^51–53^. The reflectance of surfaces covered with protruding structures, which in the case of plants includes the ultrastructure of epicuticular wax, depending on the type of structure, its size, and the density of crystal arrangement^54^. According to the literature, the surface of leaves covered with a filamentous wax structure is characterized by higher reflectance than those included in the platelet class^51^. It is indicated as an important protection against excess radiation that can damage the photosynthetic apparatus, which occurs simultaneously with drought stress. In this context, the appearance of new clustered filament structures in the wax layer of the GL 811 line and protruding tubule stacks in the GL RXL10 line could indicate the emergence of different adaptive mechanisms in these lines to the prevailing drought conditions.

### Effect of wax cover type and drought on flag leaf hydration (LH) and wax amount (WA)

Another adaptive mechanism during drought is wax accumulation, often associated with superior tolerance to drought^22,25,55^. The accumulation of epicuticular wax protects against water loss by reducing cuticular transpiration^56,57^, a lower value of which may be a functional advantage under water deficit conditions, enabling more efficient use of available water. So far, GL lines have been indicated as lines characterized by higher leaf WA under drought conditions in comparison with N-GL lines^17,22^. On the contrary, studies by Laskoś et al.^24^ on rye NILs showed no clear difference between GL and N-GL lines, suggesting that this is a more complex issue related to the mechanism of wax deposition under different environmental conditions. In the current research, the GL lines showed no clear tendency in wax accumulations under both treatments, and WA was highly dependent on the tested NIL pair. Similarly to Laskoś et al.^24^, no clear link between glaucousness and higher WA was reported, and WA was closely related to genotype and environmental conditions. Studies on wheat NILs reported wax accumulation under drought conditions in both GL and N-GL lines, the increase in wax load was between 5 and 26% or even more than two times, but the differences in the percentage increase in wax load between the N-GL and GL lines were little^17,22^. Our research also indicated that in the context of wax accumulation on leaves, the responses of N-GL and GL plants are genotype-dependent. There are reports of the potential involvement of wax biosynthesis transcription factors in other biosynthetic pathways that may be related to physiological and also biochemical responses of plants to drought stress^26,58^. In this context, the differential wax deposition on leaves of the GL and N-GL lines may also be related to different drought responses and their severity. Consistent with the study by Su et al.^17^ on wheat NILs, the drought-induced changes in wax accumulation on the leaves of the GL and N-GL lines of rye did not correlate with LH. Therefore, the relationship between the flag leaf LH and the WA is not straightforward. Nevertheless, wax accumulation could be related to other physiological and biochemical parameters via the relationship between wax biosynthesis pathways and the activation of other drought reactions.

### Effect of wax cover type and drought on stomatal conductance (*g*_s_) of flag leaf

Stomatal conductance (*g*_s_) is a substantial process and expresses carbon accumulation and transpiration in plants through the stomata, with CO_2_ flowing into the intercellular space to photosynthesis sites^59^. Consistent with the often reported in the literature decrease in *g*_s_ under drought^59–61^, stomatal conductance in rye NILs appeared to be sensitive to soil drought stress and was reduced regardless of glaucousness, particularly in the 811/811bw pair and also to a lesser extent in the RXL10/RXL10bw pair (Fig. 3). This may be related to changes in the plant’s water management strategy under drought conditions to water-saving when a stress signal reaches the leaves^62–64^. The dual limitation of water loss in the N-GL RXL10bw line via non-stomatal (high wax load) and stomatal adjustment was especially interesting (Figs. 2, 3). Despite the varied trends in wax accumulation during drought, strongly limited *g*_s_ was indicated by stomatal restrictions, which may be related to using the internal pool of CO_2_ rather than atmospheric sources. Similarly to the current study, wheat NILs studies^17,22^ also showed no clear relationship between wax accumulation and *g*_s_; despite the massive increase in leaf wax load of the GL and N-GL lines, differential sensitivity and regulation of *g*_s_ were reported. Our results may indicate that neither glaucousness nor a large amount of accumulated wax did not directly determine the regulation of the stomatal opening and closing mechanism under drought but could be related to wax biosynthesis and its regulating factors.

### Effect of wax cover type and drought on chlorophyll *a* fluorescence (CF) parameters of flag leaf

As reported by Sheperd and Griffiths^51^, wax accumulation and modification of leaf surface topography during a prolonged water deficit could affect the activity of photosystems and changes in plant productivity as a result of drought and accompanying photodamage. Few reports involve the analysis of CF parameters in GL and N-GL lines under drought conditions^22,24^. The results obtained in the present investigation, similar to reports by Guo et al.^22^ and Laskoś et al.^24^, showed varying severity of changes in CF parameters in response to drought between NILs differing in wax cover type. Consistent with reports on winter rye^24,65^, the maximum PSII photochemical efficiency was found to be insensitive to soil drought stress compared with the other CF parameters (Fig. 4). As in Laskoś et al.^24^, the photosynthetic apparatus of the 811/811bw NIL pair was much more sensitive to drought than the other NILs (Fig. 4); the overall performance index of PSII (PI) dropped even about 60%, while it decreased among other NILs about 20–40% (Fig. 4), again highlighting the difference between the drought resistance of the tested rye NILs. The N-GL 811bw line revealed more substantial drought-induced damage that affects light energy absorption (ABS/CS_m_), the number of active reaction centers (RC/CS_m_), energy trapped in reaction centers (TR_o_/CS_m_) and its intake for electron transport (ET_o_/CS_m_), but eventually the PI decline in the GL and N-GL lines was similar and amounted to 55.8 and 60.1%, respectively (Fig. 4a). This could be related to the more prominent reduction in the energy dissipated from PSII (DI_o_/CS_m_) by the N-GL 811bw line compared with the GL line, which indicated an attempt to balance the losses in absorbed energy. Enhanced photoprotection in plants exhibiting glaucousness is associated with up to 30% or higher reflectance than in N-GL lines^66,67^. These reports support the idea that the highly dense network of tubular crystals that constitute the cover of the GL 811, L35, and RXL10 lines (Fig. 1) contributed to less damage in the GL lines compared with their N-GL counterpart, for example, reflected in less reduction in PI. However, considering the highest impairment of PSII performance by the 811/811bw pair, it is substantial to examine differences between the GL and N-GL lines in pairs. In the N-GL RXL10bw line, despite the more pronounced reduction in PI in response to drought reported compared with the GL RXL10 line, the remaining CF parameters (ABS/CS_m_, TR_o_/CS_m_, ET_o_/CS_m_) had a lower percentage reduction than in GL NIL. The advantage of the GL RXL10 line in terms of less PI reduction could result from minimizing the dissipated energy (DI_o_/CS_m_), which remained unchanged in the N-GL RXL10bw line (Fig. 4). However, high WA in the N-GL RXL10bw line did not protect it from PI drop under drought. Therefore, the massive accumulation of wax did not directly indicate more substantial protection of PSII against photodamage under drought, evidenced by the contrasting deposition mechanisms reported in the current study. However, genes and regulatory factors involved in the wax biosynthesis pathway during drought can activate other biosynthesis and regulatory pathways and thus could be associated with different physiological responses important for improved drought resistance^25^.

### Effect of wax cover type and drought on flag leaf greenness index (LGI)

The LGI is a spectrophotometrically measured index of chlorophyll content in SPAD units. There are reports on a positive correlation between leaf chlorophyll content and LGI^68^. However, LGI should not be interpreted as the absolute chlorophyll content of leaves because it is sensitive to the heterogeneous distribution of molecules in the leaf^69^. The literature is scarce in reports on differences in LGI in the context of glaucousness. In the current study, drought reduced LGI in the leaves of rye NILs, except for the N-GL RXL10bw line. There are studies on the decrease in LGI in response to conditions of limited water availability^70^ that cause oxidative stress leading to chlorophyll degradation^71^. According to Silva et al.^72^, a pronounced decrease in LGI is observed in lines more susceptible to drought, which would be consistent with the results obtained in lines of the 811/811bw pair, which experienced the most substantial decrease in LGI in response to drought (Fig. 5). The lack of changes in LGI under drought conditions in the N-GL RXL10bw line may indicate the existence of defence mechanisms of this line against the significant oxidation of photosynthetic pigments.

### Effect of wax cover type and drought on malondialdehyde (MDA) content in flag leaf

The N-GL RXL10 line also showed the highest increase in MDA in the flag leaf (Fig. 6). Surprisingly, despite the reduction in PSII efficiency in the N-GL RXL10bw line, although not the greatest compared to the 811/811bw pair, it did not experience a decrease in the LGI (Fig. 5) and maintained a stable yield (Fig. 7). The accumulation of MDA could be an expected response in drought-treated plants since MDA is formed during damage to fatty acids in cell membranes and is an indicator reflecting the degree of membrane lipid peroxidation^73^. Drought was shown to be a factor inducing the accumulation of MDA^24,73,74^. However, there are reports of transient induction of MDA-induced abiotic stress genes (dehydration or heat-related genes) and subsequent activation of the antioxidant system. Under certain conditions at high cytosolic pH, the chemical reactivity of MDA is low, and the delivery of large amounts of MDA can indicate an adaptive mechanism, i.e., MDA being like a signal for gene expression of enzymes and antioxidant molecules during oxidative stress^75–77^. In this context, the high MDA in the N-GL RXL10 line could have been transient, and further investigation of this phenomenon in the N-GL RXL10 line using an appropriate approach is required^75,76^.

### Effect of wax cover type and drought on thousand grain weight and grain number

Long-term water deficiency can affect the dissolution of nutrients and their transport, turgor pressure in tissues, photosynthesis and respiration, temperature regulation, and biochemical processes in plants. Eventually, the quantity and quality of crop yield are lowered^3,65,78^. Interestingly, in response to drought, the TGW in the N-GL RXL10bw line increased by about 7% and the grain number remained unchanged (Fig. 7a,b), which also supports the theory concerning another role of MDA in this line or the appearance of other drought resistance mechanisms leading to stable yield under drought. Its counterpart, the GL RXL10 line, produced significantly fewer seeds after drought treatment, but TGW was stable, so seed quality did not deteriorate; a similar pattern can be seen for the lines of the L35/L35bw pair (Fig. 7a,b). On the contrary, the 811/811bw NIL pair produced much fewer and lighter seeds, as evidenced by a decrease in TGW under drought (Fig. 7a,b). The pronounced differences in drought resistance mechanisms between GL and N-GL lines were only present in the case of the RXL10/RXL10bw pair.

### Correlation between yield-based drought resistance indices and physiological-biochemical changes under drought in near-isogenic lines differing in wax cover type

The above was also confirmed by the results of the PCA analysis regarding the reaction to soil drought in the studied GL and N-GL NILs of rye. The PCA concerned the drought-to-control percentage ratio of physiological and biochemical parameters and the yield-based drought resistance indices. Some reports concerning the PCA analysis highlight the differential classification among cereal genotypes in terms of resistance to drought and enable the development and testing of hypotheses, as well as the understanding of complex responses to applied treatment^46,60,79^. The GL RXL10 and N-GL RXL10bw lines were located far apart in the PCA biplot, while the opposite relationship was observed for the 811/811bw and L35/L35bw NIL pairs (Fig. 8). These differences occurred primarily due to the prominent increase in WA and MDA in the N-GL RXL10bw line and the higher yield stability of this line compared with its GL counterpart (Fig. 8). This could indicate different drought resistance mechanisms in the GL and N-GL lines in the RXL10/RXL10bw NIL pair, which was not evident in other NILs pairs. The position of the pair of GL 811 and N-GL 811bw lines (high DSI, TOL, SDI values) indicated high susceptibility to drought compared with the other NILs. The opposite position of the L35/L35bw NIL pair suggests its high resistance to drought, which is consistent with the observations for this line presented by Laskoś et al.^24^.

## Conclusions

The results reported here confirm that the wax load accumulation cannot be directly correlated with the plant's response to soil drought stress. The presented study showed a differentiated reaction to drought among tested NILs in each pair, the most prominent differences in responses to water deficit were observed between GL and N-GL lines of the RXL10/RXL10bw NIL pair.. Particular attention should be paid to the grain number and stable thousand grain weight of the N-GL RXL10bw line under soil drought conditions and accompanying reactions, such as about a 60% increase in MDA and about a two-fold increase in wax amount, both of which were notably higher compared with its counterpart the GL RXL10 line and other rye NILs. In the GL RXL10 line, the appearance of tubular clusters on its abaxial leaf surface is noteworthy, which could contribute to protection against photodamage. This line maintained a stable thousand grain weight but eventually did not avoid a decline in seed number in response to drought. So far, the pair RXL10/RXL10bw of rye lines showed the most significant differences in drought responses and will be a suitable object for further research on the relationship between glaucousness and drought resistance. We concluded that the investigation performed on rye NILs suggests that a line-specific drought resistance could be associated with the wax biosynthetic pathways involved in physiological and biochemical responses.

## Supplementary Information

Supplementary Information.

## Data Availability

All data analysed during this study are included in this published article and its supplementary information file.

## Abbreviations

NILs: Near-isogenic lines
GL: Glaucous
N-GL: Non-glaucous
CF: Chlorophyll *a* fluorescence kinetics
MDA: Malondialdehyde
SEM: Scanning electron microscope
FW: Fresh weight
DW: Dry weight
LH: Leaf hydration
LGI: Leaf greennesss index
PCA: Principal component analysis
WA: Wax amount
