# Supplementary material for: Variation between glaucous and non-glaucous near-isogenic lines of rye (Secale cereale L.) under drought stress
Source: Sci Rep. 2022 Dec 28;12:22486. doi: 10.1038/s41598-022-26869-6 (PMC9797576; doi:10.1038/s41598-022-26869-6)
Supplement: Supplementary file 1 — Supplementary Information. [file 41598_2022_26869_MOESM1_ESM.pdf]

Supplementary Information

Tab. S1. Mean values ±SE (n=7) of chlorophyll *a* fluorescence kinetic parameters (F_v/F_m, Area, PI, ABS/CS_m, TR_o/CS_m, ET_o/CS_m, DI_o/CS_m, RC/CS_m) in glaucous (GL) and non-glaucous (N-GL) near-isogenic lines (NILs) of rye under control (C) and drought (D) conditions. The statistically significant differences between the means in control and drought were verified for P ≤ 0.001(***), P ≤ 0.01(**),P ≤ 0.05(*) according to Student's test. ns, not significant. SE, standard error. *T, treatment;* F_v/F_m, *the maximum photochemical efficiency of photosystem II (PSII); Area, the size of the electron acceptor field of PSII; PI, the overall performance index of PSII;* ABS/CS_m, *the photon flux absorbed by antenna dye molecules;* TR_o/CS_m, *the excitation energy trapped in PSII reaction centers;* ET_o/CS_m, *the electron transport rate by PSII;* DI_o/CS_m, *the energy dissipated from PSII;* RC/CS_m, *the density of active reaction centers.*

NILs	*T*	*Fv/Fm*		*Area*		*PI*		*ABS/CSm*		*TRo/CSm*		*ETo/CSm*		*DIo/CSm*		*RC/CSm*	
GL 811	C	0,820±0,001	***	30171±1396	***	4,32±0,16	***	1295±33	**	1063±28	**	674±22	***	232±5	**	704±15	***
	D	0,801±0,004		17300±1994		1,91±0,34		1005±86		805±73		437±53		200±14		398±55	
N-GL 811bw	C	0,820±0,003	*	24233±1961	*	3,75±0,45	*	1157±37	**	950±34	**	582±35	**	208±4	***	588±39	**
	D	0,796±0,01		13200±2386		1,49±0,42		796±71		635±65		325±54		161±6		281±51	
GL L35	C	0,798±0,004	ns	27433±2471	ns	3,72±0,49	ns	1220±37	ns	973±34	ns	591±38	ns	247±4	ns	726±48	ns
	D	0,796±0,002		24320±2446		3,03±0,39		1120±59		892±47		524±43		229±12		604±55	
N-GL L35bw	C	0,803±0,003	ns	27175±847	**	3,83±0,17	**	1156±29	ns	929±25	ns	571±22	*	227±4	ns	679±24	***
	D	0,797±0,002		21343±1426		2,45±0,34		1085±22		864±19		490±26		220±3		502±32	
GL RXL10	C	0,794±0,002	ns	30967±618	*	3,9±0,17	**	1176±20	**	934±17	**	573±15	**	242±5	*	745±19	***
	D	0,786±0,003		23833±1896		2,65±0,34		1017±31		799±24		469±28		218±8		507±36	
N-GL RXL10bw	C	0,799±0,005	**	26080±2492	*	3,29±0,43	**	1044±40	ns	834±32	*	483±36	ns	210±10	ns	610±39	**
	D	0,775±0,002		20300±1087		1,99±0,07		958±20		743±15		418±13		216±5		428±13	

Tab. S2. Drought resistance indices in glaucous (GL) and non-glaucous (N-GL) near-isogenic lines (NILs) of rye, that is, the drought to control percentage ratio (D/C_{GW} and D/C_{TGW}) and the drought susceptibility index (DSI_{GW} and DSI_{TGW}) calculated based on grain weight (GW) and thousand grain weight (TGW), and indices calculated based on grain weight, that is, tolerance index (TOL), Mean Productivity (MP), Harmonic Mean (HM), Geometric Mean Productivity (GMP), Yield Index (YI), Sensitivity Drought Index (SDI), Yield Stability Index (YSI).

NILs	***D/C_{GW}***	***D/C_{TGW}***	***DSI_{GW}***	***DSI_{TGW}***	***TOL***	***MP***	***HM***	***GMP***	***YI***	***YSI***	***SDI***
GL 811	25,84	78,23	1,42	3,55	0,26	0,22	0,14	0,18	0,75	0,26	0,74
N-GL 811bw	21,38	72,35	1,51	4,51	0,27	0,21	0,12	0,16	0,62	0,21	0,79
GL L35	74,46	104,29	0,49	-0,70	0,06	0,22	0,21	0,22	1,54	0,74	0,26
N-GL L35bw	78,60	98,16	0,41	0,30	0,05	0,21	0,21	0,21	1,53	0,79	0,21
GL RXL10	30,40	102,18	1,34	-0,36	0,15	0,14	0,10	0,12	0,53	0,30	0,70
N-GL RXL10	104,64	107,02	-0,09	-1,14	-0,01	0,12	0,12	0,12	1,03	1,05	-0,05
